# Model for in-vivo estimation of stiffness of tibiofemoral joint using MR imaging and FEM analysis

**DOI:** 10.1186/s12967-021-02977-1

**Published:** 2021-07-19

**Authors:** Sandeep Panwar Jogi, Rafeek Thaha, Sriram Rajan, Vidur Mahajan, Vasantha Kumar Venugopal, Anup Singh, Amit Mehndiratta

**Affiliations:** 1 Centre for Biomedical Engineering, Indian Institute of Technology, Delhi, New Delhi 110016 India; 2 Amity University Haryana, Gurgaon, 122413 India; 3 Mahajan Imaging Centre, New Delhi, 110016 India; 4 Department of Biomedical Engineering, All India Institute of Medical Sciences, New Delhi, 110029 India

**Keywords:** Biomedical engineering, Finite element analysis, Magnetic resonance imaging, Solid modeling

## Abstract

**Background:**

Appropriate structural and material properties are essential for finite-element-modeling (FEM). In knee FEM, structural information could extract through 3D-imaging, but the individual subject’s tissue material properties are inaccessible.

**Purpose:**

The current study's purpose was to develop a methodology to estimate the subject-specific stiffness of the tibiofemoral joint using finite-element-analysis (FEA) and MRI data of knee joint with and without load.

**Methods:**

In this study, six Magnetic Resonance Imaging (MRI) datasets were acquired from 3 healthy volunteers with axially loaded and unloaded knee joint. The strain was computed from the tibiofemoral bone gap difference (ΔmBGFT) using the knee MR images with and without load. The knee FEM study was conducted using a subject-specific knee joint 3D-model and various soft-tissue stiffness values (1 to 50 MPa) to develop subject-specific stiffness *versus* strain models.

**Results:**

Less than 1.02% absolute convergence error was observed during the simulation. Subject-specific combined stiffness of weight-bearing tibiofemoral soft-tissue was estimated with mean values as 2.40 ± 0.17 MPa. Intra-subject variability has been observed during the repeat scan in 3 subjects as 0.27, 0.12, and 0.15 MPa, respectively. All subject-specific stiffness-strain relationship data was fitted well with power function (R^2^ = 0.997).

**Conclusion:**

The current study proposed a generalized mathematical model and a methodology to estimate subject-specific stiffness of the tibiofemoral joint for FEM analysis. Such a method might enhance the efficacy of FEM in implant design optimization and biomechanics for subject-specific studies.

*Trial registration* The institutional ethics committee (IEC), Indian Institute of Technology, Delhi, India, approved the study on 20th September 2017, with reference number P-019; it was a pilot study, no clinical trail registration was recommended.

**Supplementary Information:**

The online version contains supplementary material available at 10.1186/s12967-021-02977-1.

## Background

The finite–element-modeling (FEM) with appropriate structural and biomechanical information is an efficient tool for analyzing the biomechanical behavior of the knee joint [1–4]. The structure and alignment of a bone could be accessed non-invasively using any 3D imaging techniques; however, it is still challenging to non-invasively access the soft-tissue material properties of the knee joint. Thus, to use the FEM tool for appropriate knee joint analysis, a noninvasive method is required to estimate the subject-specific knee joint soft-tissue material properties.

In literature, subject-specific 3D-model have been included in FEM without any subject-specific soft-tissue material properties [4–10]. It could be mainly due to the inaccessibility of noninvasive measurement techniques, or maybe invasive techniques [11] are hardly appreciated for such clinical research studies. Whereas, a few noninvasive techniques [12, 13] are available, such as radiography, Computed Tomography(CT), and Magnetic Resonance Imaging(MRI), which can indirectly measure biomechanical features; however, the output of these techniques do not suffice to incorporate in the FEM of the knee joint, which requires tissue mechanical properties. Other non-conventional imaging methods have reported significant changes in the knee joint MRI parameters under load conditions [14–21]. Such practices provide only an indirect indicator of the knee joint soft-tissues properties but cannot measure the stiffness or relevant biomechanical properties used for FEM analysis.

Subject-specific, tibiofemoral weight-bearing soft-tissues (WB-ST) material properties for appropriate FEM requires because (i) a significant variation in stress distribution with variation in stiffness were observed in the tibiofemoral compartment during FEM [22], and (ii) *inter*-subject [23] and *intra*-subject variability in cartilage stiffness have been reported in the past [24, 25]. Therefore, for a close approximate solution of the knee FEM, subject-specific WB-ST material properties are required.

The knee joint articular soft-tissues bear the weight, transfer the load, and provide the frictionless surface for articular motion [26]. Cartilage, meniscus, synovial fluid, supporting ligament, and muscles collectively contribute to the functioning of the knee joint [26] at the in-situ condition. However, in the literature, biomechanical properties of cartilage [24, 25, 27–32], and meniscus [32] were studied as an individual components. Whereas, for in-situ modeling of the knee joint, consideration of the collective properties of all WB-ST and the joint fluid may yield a better FEM analysis for clinical insight rather than the individually considered component properties.

The current study proposed a novel method to obtain combined-compressive-stiffness (CCS) of the tibiofemoral joint for FEM, which includes stiffness because of all tibiofemoral WB-ST (cartilage, meniscus, along with the effect of supporting tissues such as muscles and ligament) and synovial fluid. The first goal was to obtain experimentally yield subject-specific strain at the tibiofemoral joint using an axial knee joint loading device during MR imaging. Secondly, to build a subject-specific 3D-model of the knee joint using MRI data. Thirdly, subject-specific knee FEM was analyzed with various tibiofemoral joint stiffness for understanding a strain-stiffness characteristic. Then subject-specific stiffness was estimated using the individual strain-stiffness characteristic and experimentally yield strain. Thus, this study proposes a generalized mathematical model for compressive stiffness *versus* strain for the tibiofemoral joint of healthy subjects'.

## Methods

**Fig. 1:**
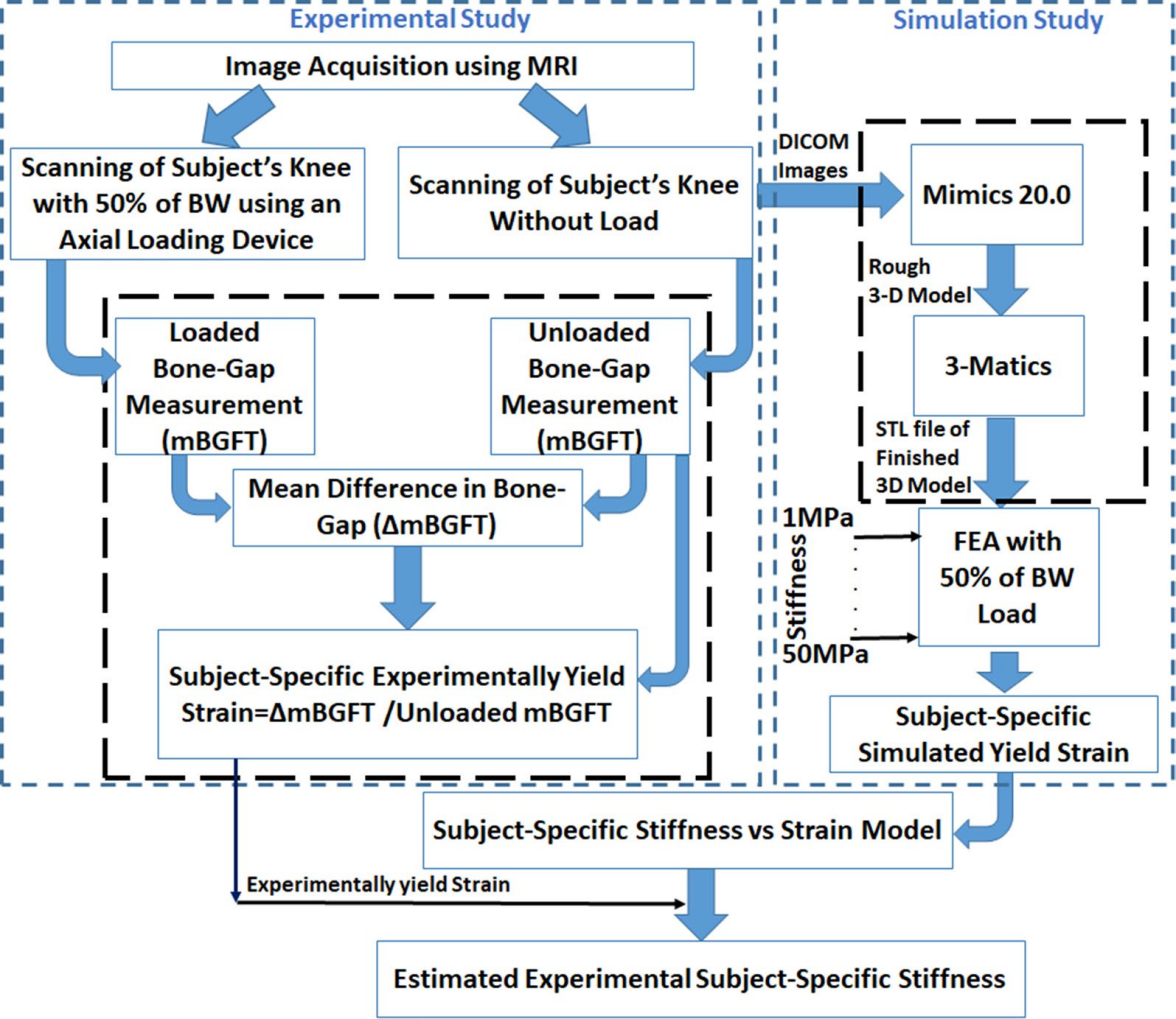
The stepwise workflow of the study is presented in the figure; Figure shows two phases of the study: experimental and Simulation; mBGFT is the mean tibiofemoral bone gap, ΔmBGFT is the difference of unloaded and loaded mBGFT

The current study enrolled three healthy male subjects, with no prior reported knee surgery, pain, or any other symptoms of knee ailment (Age: 30–35 years, Weight: 68–80 kgs, and Height: 1.65–1.73 m), for an MRI experiment with prior approval from Institutional Ethics Committee (IEC) and informed written consent of the subjects. The right leg of each subject was scanned. All volunteers were scanned twice to evaluate experimental repeatability. MR image acquisition was performed at Mahajan Imaging Centre, India, using a 3.0 Tesla MR scanner (General Electric Healthcare, Chicago, Illinois) and an 8-channel transmitter/receiver knee coil. The current study workflow and steps are presented in Fig. 1.

In the experimental study, the mean strain was computed at the tibiofemoral joint. A simulation study was carried out to develop a subject-specific mathematical model of the strain-stiffness relationship. Finally, subject-specific stiffness was estimated using experimentally yielded subject-specific mean strain with the subject-specific mathematical model, as shown in Fig. 1. In the experimental study, Fig. 1 shows the intermediate steps of data processing described below:

*Step-1:* MR image acquisition of the knee joint under load and without load.

*Step-2:* Measurement of mean tibiofemoral bone gap (mBGFT) for both loaded and unloaded knee joint.

*Step-3:* Calculation of difference of mBGFT of unloaded and loaded condition (ΔmBGFT) to measure experimental yield mean strain at tibiofemoral joint.

In the simulation study, Fig. 1 shows the following steps:

*Step-1:* Development of rough 3D-model in MIMICS using unloaded scanned knee joint.

*Step-2:* Development of smoothened 3D-model.

*Step-3:* Finite-Element-Modeling.

### MRI compatible axial loading device

A custom-built MRI-compatible loading device (details in Additional file 1: Sect. 1), validated with standing open-MRI, was used in the current study. The device was designed to apply the load between the waist and foot sole, bi-directionally opposite to each other. Images were acquired without load and with 50% of body-weight (50%BW) of the individual subject using this MRI-compatible axial loading device.

### Image acquisition

In the current study, MR images were acquired using 3D-Fast Spoiled Gradient Echo (FSPGR) Fat Saturated sequence (repetition Time (TR) = 10.8 ms, echo time (TE) = 3.5 ms, reconstructed image size = 512 × 512 pixels; the field of view = 140 mm × 140 mm; slice thickness = 2 mm; number of slices = 72; filp angle = 5; pixel bandwidth = 61 Hz/pixel).

Subjects were relaxed for an hour before scanning to avoid any prior loading effect. Images were acquired without a load on the knee joint, and immediately after, subjects were scanned with a load of 50%BW using the MRI-compatible axial loading device. For the repetitive study, all subjects were re-scanned after a gap of atleast 1 day.

### Bone gap measurement

A semi-automatic, in-house-built routine measured the tibiofemoral bone gap using MATLAB R2018a (The MathWorks Inc., Natick, MA, USA). The segmented regions were validated by an expert radiologist (with 16 years of experience in Musculoskeletal Radiology). The bone gap was measured by selecting a region of interest (RoI) on knee joint images containing trochlea until the femoral condyles containing cartilage [16]. The bone gap was measured by minimum Euclidian distance between the distal surface of the femur and the proximal surface of the tibia at each slice. The mean distance computed was observed as a mean bone gap between the femur and tibia (mBGFT).

Change in the tibiofemoral mean bone gap (ΔmBGFT) was calculated as the difference between without load mBGFT and with load mBGFT, as shown flow-chart in Fig. 1.

### Development of 3D-model of the knee joint

#### DICOM images to 3D-surface model

**Fig. 2:**
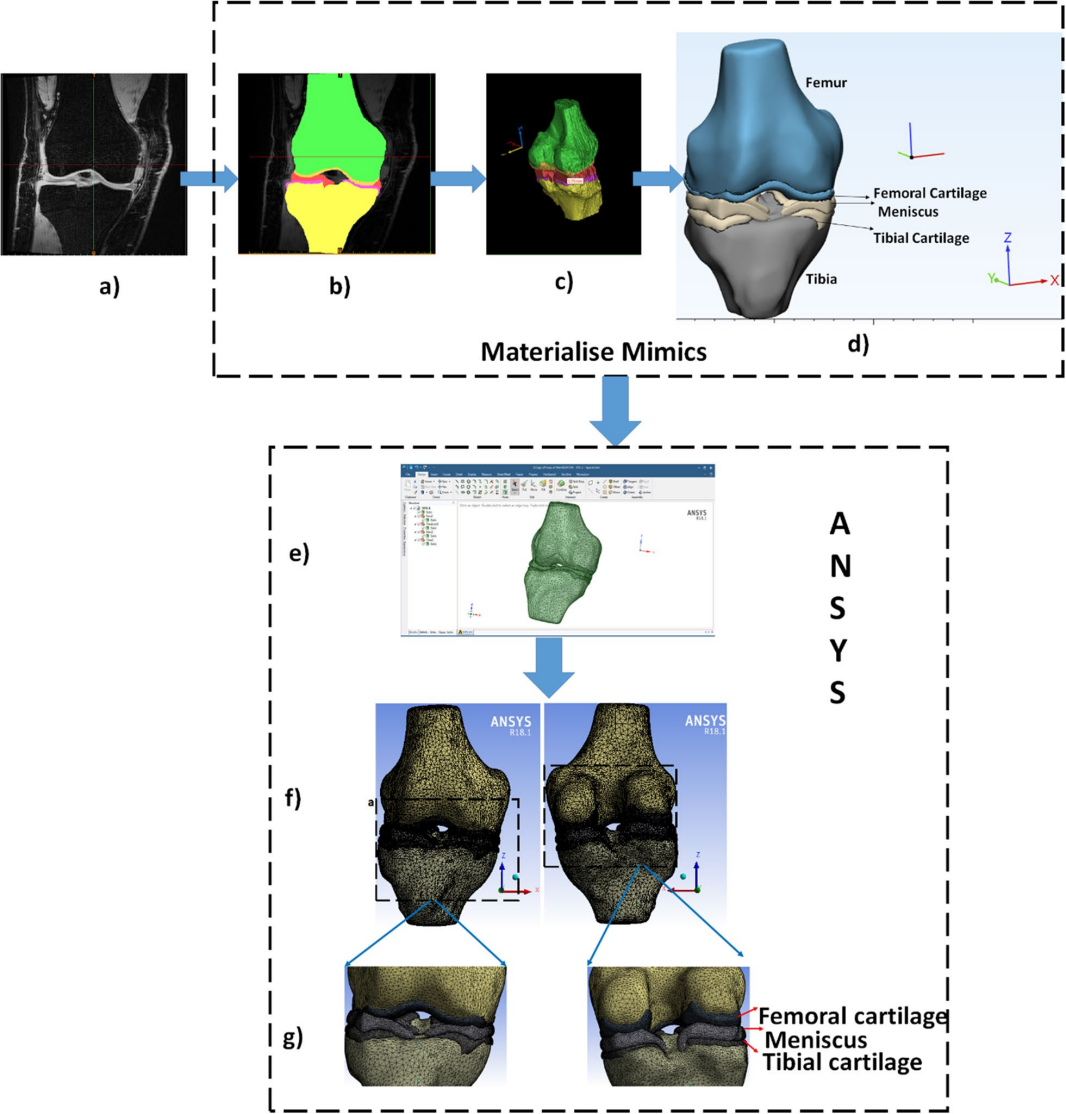
The stepwise processing of data. **a** representative slice of 3D-FSPGR image of knee joint, **b** segmented tissues (cartilage, bone, and meniscus) and overlaid on the grayscale MRI image, **c**, **d** development phase of 3D surface geometry at Mimics 20.0 and 3-Matic Research 12.0, respectively, **e** SpaceClaim platform used to convert STL files in the solid CAD model and assembly formation. **f** Anterior and posterior view of mesh model. **g** Zoom-in anterior and posterior view to visualize the cartilage and meniscus, tibiofemoral bone gap, ΔmBGFT is the difference of unloaded and loaded mBGFT

Acquired MRI images in DICOM format (as shown in Fig. 2a) of each subject were imported in MIMICS Research 20.0 (Materialise NV, Leuven, Belgium). The femur, femoral-cartilage, tibia, tibial-cartilage, and meniscus were manually segmented (shown in Fig. 2b) and validated by the same radiologist to develop 3D-surfaces geometries as the example shown in Fig. 2c. Before further processing, morphological operations were performed. The prerequisite workflow to develop FEM compatible with 3D-surface geometry is shown in Fig. 1 and with the example in Fig. 2.

Further, the developed 3D-geometries were smoothened using the Laplacian first-order method with a small smoothing factor [7] of 0.3, using 3-MATIC Research 12.0 (Materialise NV, Leuven, Belgium), as shown in Fig. 2c (detail in Additional file 1: Sect. 2a). The final models were imported as STL files of each component in Ansys 18.1 Workbench (Ansys Inc., Canonsburg, Pennsylvania, United States). Before FEM, all geometries were further optimized in SpaceClaim to assemble their own coordinate space, as shown in Fig. 2e (detail in Additional file 1: Sect. 2b).

### Finite-element-analysis (FEA)

#### Material properties

In this study, the Isotropic elastic (*IE*) model [5] of the material property was deployed to reduce the computational complexity. 'Engineering Data' component of Static Structural was fed with ten WB-ST stiffness values as 1, 2, 3, 5, 10, 15, 20, 25, 30, and 50 MPa. Poisson's ratios were used as 0.45 and 0.3 for cartilage and meniscus, respectively [33]. However, Young’s Modulus and Poisson's ratio of bone was kept constant as 1000 MPa and 0.3, respectively [9] for all simulations.

#### Assigning contacts and meshing

**Fig. 3:**
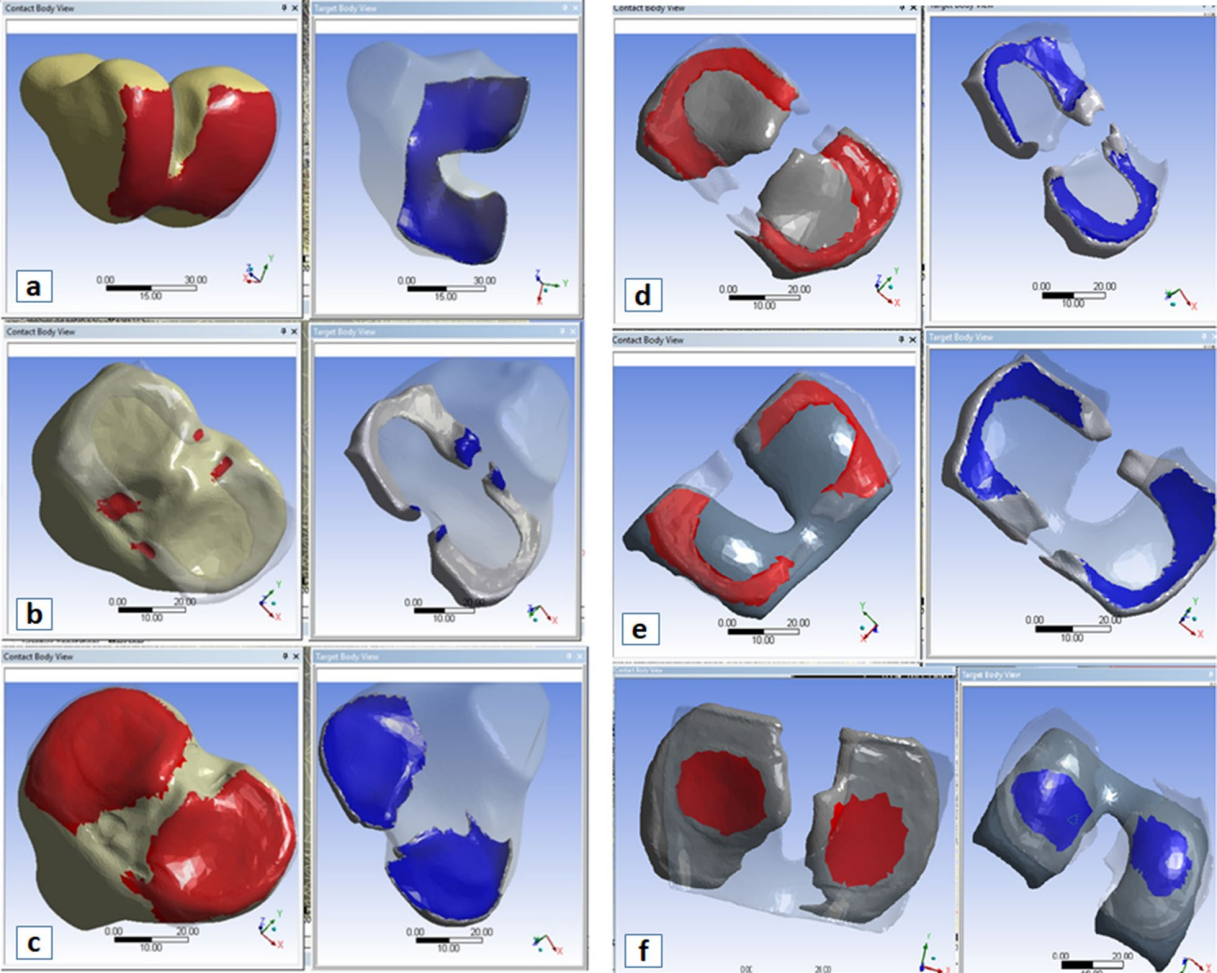
**a**–**c** show bonded contacts, where **a** femur with femoral-cartilage, **b** tibia with meniscus and **c** tibia with tibial-cartilage; Left-hand side shows contact bodies and the right-hand side shows targeted bodies as opaque; **d**–**f** shows frictional contacts, **d** tibial-cartilage with the meniscus, **e** femoral-cartilage with meniscus and **f** femoral-cartilage with tibial-cartilage; Left-hand side shows contact bodies and the right-hand side shows targeted bodies as opaque

Further, multi-point constraint (MPC) contact formulation was used for the solution in bonded contacts (interfaces at femur with femoral cartilage, tibia with tibial cartilage, and tibia with meniscus), as shown in Fig. 3a–c. Augmented Lagrange (*AL*) formulation was used for all frictionless contacts (interfaces at femoral-cartilage with tibial-cartilage, femoral-cartilage with the meniscus, and tibial-cartilage with meniscus), as shown in Fig. 3d–f (Detail in Additional file 1: Sect. 3a). All components of the model were meshed with TET10 configuration for providing an enhanced formulation for better fitting and less computational complexity [34, 35] (Detail in Additional file 1: Sect. 3b).

The range of the number of nodes and elements for soft tissues (cartilage and meniscus) was 120 K–170 K and 70 K–110 K, respectively. However, the range of the number of nodes and elements for bone was 50 K–65Ks and 32 K–38 K, respectively. The number of elements and nodes of soft tissues (cartilage and meniscus) was larger than the bone because the soft-tissues' mesh size was four times lesser than the bone's mesh size (details in Additional file 1: Sect. 3b).

#### Applied load and boundary conditions

The model's orientation was such as articulation surfaces are in the x–y plane, and tibia and femur bone shaft are in the z-direction. A remote force of *50%BW* was simulated on the femur in z-direction toward the tibia in five substeps with 80 N increment. Substep loads are gradually applied to facilitate the convergence. Fixed support was provided to the distal surfaces of the tibia. Further, a remote displacement was applied to the femur with *Z*-direction free and rotation 0^◦^ in all directions (assuming no rotational movement at full extension knee).

#### Solver algorithm

The numerical criterion chosen to evaluate the mesh density was Directional Deformation. Numerical implementation conducted as the Static Structural. During the load-step implicit iterative preconditioned conjugate gradients (PCG) solver was used. “Large deflection” was implemented only for WB-ST stiffness value < 5 MPa. The full Newton–Raphson solution procedure was used for non-linear analysis. An adaptive convergence was conducted by allowing a maximum convergence error of 5%. The convergence error of each simulation was reported to evaluate the mesh-dependent error. This method adaptively refined the mesh in each refinement loop till the maximum allowed convergence was achieved.

The FEA simulation was conducted on a workstation with Intel(R) Xeon(R) CPU E5-2630 version3 @ 2.40 GHz dual-processor system and 64 GB random access memory. The computation time was observed to increase with a decrease in stiffness of soft-tissue.

### Data analysis

FEM solved the femoral deformation in all three directions for the various simulated values of Young’s Modulus (1 to 50 MPa) of soft-tissues for each CAD model. The simulated deformation in z-direction was due to applied load against the assigned CCS of material during the simulation. Deformation in z-direction in the femur represented the change in the bone gap between the femur and tibia because the tibia was kept fixed during the simulations, and force was applied at the femur's proximal end. This arrangement would provide different z-direction deformation at the femur's distal surface against each Young's Modulus of the soft tissues fed in the simulation. Further, corresponding to the Young’s Modulus, compressive strain (‘*ε*’) was calculated by dividing FEM simulated deformation by the unloaded mBGFT to normalize the variability of mBGFT of each dataset. Compressive stiffness *versus* FEM simulated compressive strain graph was plotted for each subject, and data were fitted using the following model equation:

1 $$\varepsilon = a*~E^{{ - b}}$$

where ‘*E*’ is compressive stiffness, and ‘*ε*’ is a strain, ‘*a*’ is the scaling coefficient representing mean stress with unit N/mm^2^, and ‘*b*’ is the unitless power coefficient. The stress–strain relationship inspires the Eq. (1) in the linear region where ‘*b*’ is equal to 1. Whereas in (1), the value of ‘*b*’ would be estimated. Subject-specific estimated CCS of the tibiofemoral joint was calculated using a subject-specific experimentally yield mean strain in a subject-specific mathematical model.

## Results

### Bone gap measurement

The mean of the unloaded mBGFT was observed as 5.99 ± 0.72 mm. Whereas the average change in mBGFT due to 50%BW was observed as 0.63 ± 0.15 mm. The measured unloaded and loaded mBGFT of all the subjects and calculated ΔmBGFT are shown in Table 1. The range of the experimental yield strain among the subjects was observed as 0.068 mm to 0.158 mm.

**Table 1:** Tibio-femoral bone gap measured without load and with load

	Subject 1	Subject 1 repeat	Subject 2	Subject 2 repeat	Subject 3	Subject 3 repeat
Unloaded mBGFT (mm)	7.31	5.64	5.35	5.92	5.22	6.54
Loaded mBGFT (mm)	6.81	5.24	4.76	4.99	4.64	5.88
ΔmBGFT (mm)	0.50	0.44	0.59	0.94	0.59	0.67
% ΔmBGFT about Unloaded mBGFT (%)	6.89	7.8	11.01	15.85	11.24	10.23

*mBGFT* mean tibiofemoral bone gap, *ΔmBGFT* mean difference of unloaded and loaded bone gap

### Finite-element-analysis

**Fig. 4:**
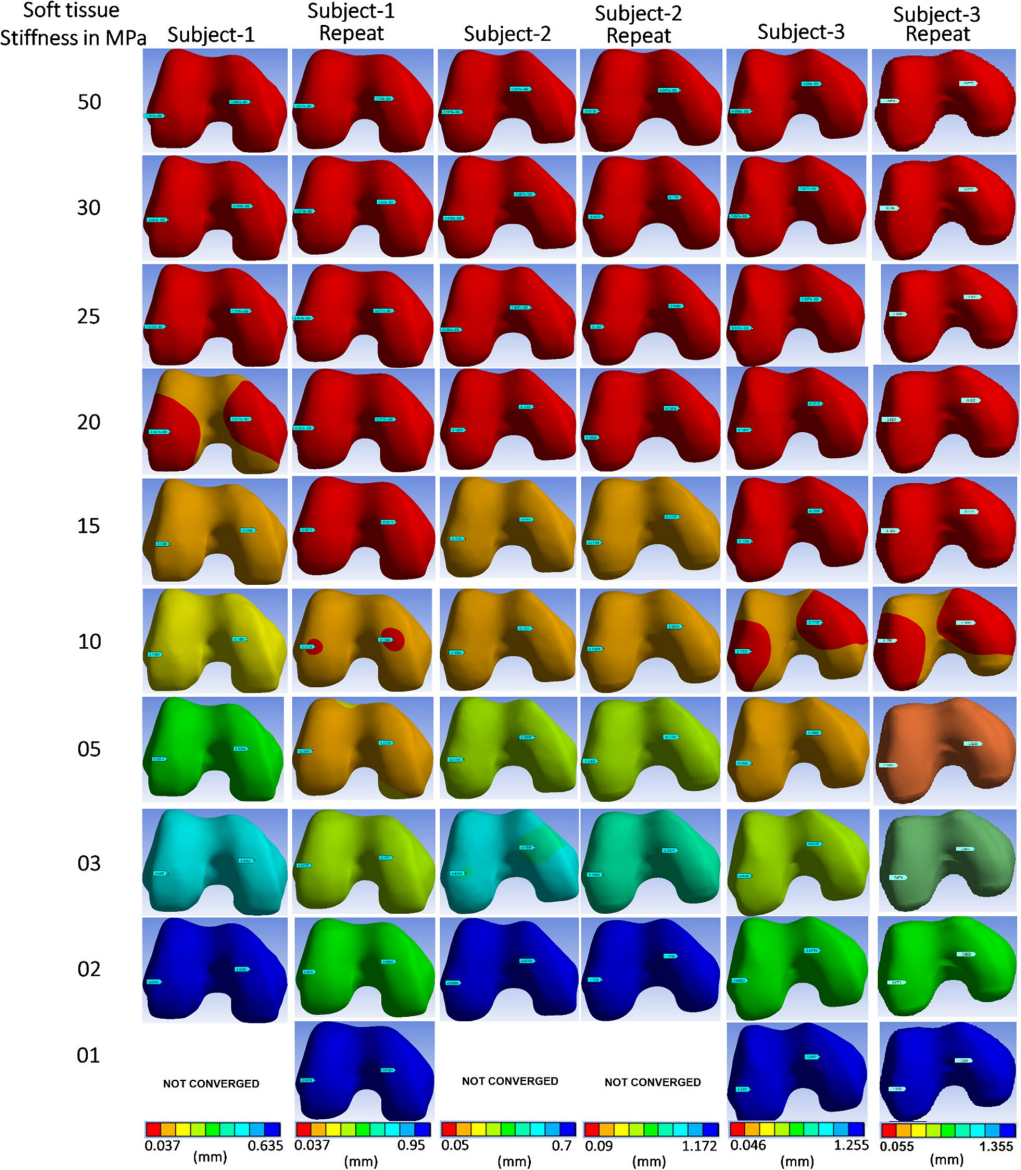
The deformation profiles of each subject with the FEM model using various soft-tissue stiffness values

**Fig. 5:**
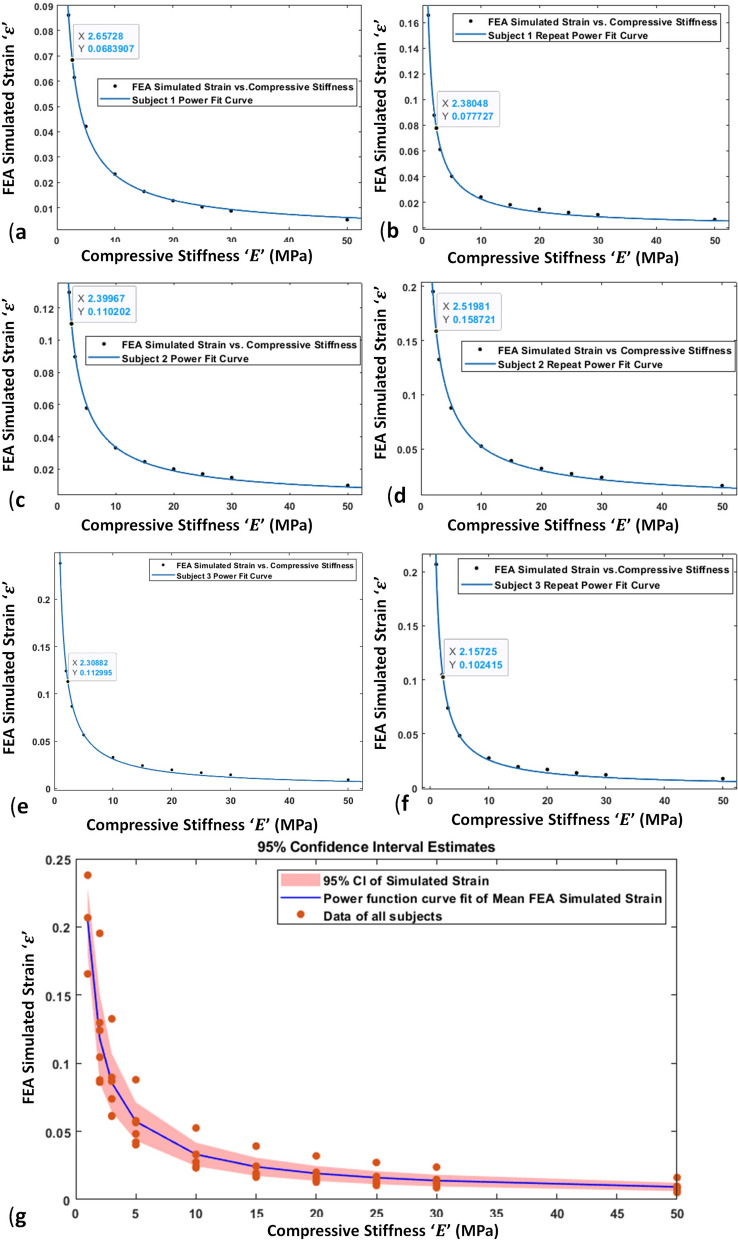
Subject-1, Subject-1 Repeat, Subject-2, Subject-2 Repeat, Subject-3, and Subject-3 Repeat power function curve fitting graph for finite-element-analysis (FEA) simulated strain *versus* compressive stiffness of weight-bearing tibiofemoral soft-tissues shows in figure **a**–**f** respectively; **g** shows 95% confidence interval estimation of all subject (All Graphs Developed in MATLAB R2018a)

FEM simulated deformation in the femur bone's distal surface was analogous to experimentally obtained ΔmBGFT using knee MRI images. The femur's deformation consistently increases with a decrease in stiffness in the range of 0.59 mm to 1.29 mm under a constant load in all FEM models, as shown in Figs. 4 and 5. Simulated results with various assigned properties of the tibiofemoral joint are shown in Table 2. In addition, a power function model was observed consistently for all subjects to define the relationship between simulated strain and stiffness, as shown in the graphs of Fig. 5.

**Table 2:** Subject specific estimated combined compressive stiffness

	Experimentally obtained ΔmBGFT (mm)	Experimentally obtained mBGFT (mm)	Calculated mean strain ε = ΔmBGFT/mBGFT	Applied Force (N) (50% Body weight)	Scaling Factor 'a' of Eq. (1) (N/mm^2^)	Power factor 'b' of Eq. (1)	Young's Modulus of soft tissue estimate by model ‘E’ (MPa)
Subject1	0.5	7.31	0.06839	400	0.1527	− 0.8223	**2.657**
Subject2 repeat	0.44	5.66	0.07773	400	0.1638	− 0.8607	**2.38**
Subject2	0.59	5.35	0.11028	340	0.2279	− 0.8315	**2.4**
Subject2 repeat	0.94	5.92	0.15878	340	0.3334	− 0.8036	**2.52**
Subject3	0.59	5.22	0.11302	350	0.2356	− 0.8794	**2.309**
Subject3 repeat	0.67	6.54	0.10244	350	0.2042	− 0.8997	**2.1572**
Mean ± SD	0.621 ± 0.17	6.000 ± 0.79	0.1051 ± 0.031		0.2196 ± 0.065	− 0.8495 ± 0.036	**2.4039 ± 0.172**

*mBGFT *mean tibiofemoral bone gap, *ΔmBGFT* mean difference of unloaded and loaded bone gap

The negative values of the convergence error percentage depict that the adaptive mesh refinement increases the deformation simulation. The average convergence error was observed − 0.186 ± 0.31% for all the simulation results, as shown in Table 3. However, the maximum error observed was − 1.012%, which is the only case where the absolute value is more than 1. The average percentage convergence error for all the subjects for FEM with Young’s modulus 2 MPa and 3 MPa was observed as − 0.056 ± 0.08 and − 0.002 ± 0.02, respectively. The computation time was observed in the range of 7–151 h. The computation time of the study increases as the assigned stiffness of soft-tissue in the FEM decreases. The FEA computation time with the soft-tissue stiffness 30 MPa and 50 MPa was a maximum of upto 16 h. However, the FEA computation time with soft-tissue stiffness 1 MPa and 2 MPa was a minimum of at least 56 h.

**Table 3:** Percentage coveregence error observed in each FEM simulation

Soft tissue stiffness in MPa	Subject 1 (%)	Subject 1 repeat (%)	Subject 2 (%)	Subject 2 repeat (%)	Subject 3 (%)	Subject 3 repeat (%)
50	− 0.113	− 0.119	0.009	− 0.235	− 1.012	− 0.975
30	− 0.002	− 0.374	− 0.076	− 0.134	− 0.860	− 0.065
25	− 0.024	− 0.023	− 0.037	− 0.082	− 0.286	− 0.328
20	− 0.278	− 0.039	− 0.016	− 0.074	− 0.196	− 0.003
15	− 0.134	− 0.749	− 0.090	− 0.828	− 0.007	− 0.613
10	− 0.480	− 0.068	− 0.006	− 0.070	− 0.025	− 0.292
5	− 0.904	− 0.429	− 0.001	− 0.048	− 0.867	− 0.101
3	− 0.005	− 0.033	− 0.258	− 0.033	− 0.003	− 0.005
2	− 0.023	0.008	− 0.003	− 0.010	0.043	− 0.027
1	NA	− 0.002	NA	NA	− 0.006	0.835

*NA* is not applicable, as simulation was not converge to solution

### Estimation of material properties (combined compressive stiffness)

Subject-specific power function model obtained with stiffness *versus* simulated strain graph, as shown in Fig. 5. The goodness of fitting (*R*^*2*^) was observed in the range of 0.997–0.999. The scaling coefficient '*a*' and power coefficient' *b*' of (1) for each subject was in the range of 0.15 to 0.33 and − 0.80 to − 0.89, respectively (as shown in Table 2.)

The estimated CCS for the subjects was observed in the range of 2.1 MPa to 2.7 MPa. The intra-subject variability observed for subject-1, subject-2, and subject-3 was 0.27 MPa, 0.12 MPa, and 0.15 MPa, respectively. Further, the results were analyzed to find a generalized relation of stiffness *versus* strain of the tibiofemoral joint in vivo in healthy subjects. A generalized mathematical model was evaluated by averaging the subject-specific coefficients (Table 2), as follow:

2 $$E = \left( {\frac{\varepsilon }{{0.2196}}} \right)^{{\left( {\frac{1}{{ - 0.8495}}} \right)}}$$

where *'ε*' is an experimental yield strain, and '*E*' is the estimated combined compressive stiffness (CCS) of the tibiofemoral joint.

## Discussions

Noninvasive methods to study the knee joint has various applications, such as for better understanding of its biomechanics [6, 7, 36, 37], tissue health [12, 15, 21, 33], cartilage degeneration [2, 16, 18, 20], weight-bearing behavior of soft-tissues [3, 5, 15, 17, 19], and further using the information for optimization of implants design [4, 22] have long been an interest of clinical research community. A noninvasive method is proposed in the current study to estimate subject-specific material properties of whole tibiofemoral knee joint as Combined Compressive Stiffness (CCS) for use in FEM. In the CCS, the effect of all the contributing soft-tissues, non-Newtonian fluids, and their dynamic interaction during load was included. A simplified finite element modeling of the knee joint was conducted in the study to estimate CCS. Further, a generalized mathematical model of the stiffness *versus* strain relationship has been presented in the study. The stiffness calculated in the current study was the collective response of all tibiofemoral joint tissue and synovial fluid since the individual componential study may be insufficient to describe the complex physical interaction of these components *in-situ* conditions.

The subject-specific model in the current study is a simplified model, which is interested in Z-directional deformation in soft-tissue because of the 50%BW load. The femur and tibial bone have various regions with different material properties, such as a medullary cavity, compact bone, and epiphysis. Whereas uniform isotropic material properties were assigned in the current study to simplify the FEM model as bone components have non-significant contributions in Z-direction deformation during the applied load.

Furthermore, other subject-specific parameters such as muscle strength, synovial fluid as non-Newtonian fluid and its pressure, and anisotropic model of cartilage and bone might be included, making the model more realistic. However, the inclusion of all these subject-specific parameters will increase the complexity in FEM and further increase computational cost. Further, the problem with using these parameters is that most of this information cannot be accessed non-invasively, thus prohibits the use of such information in real clinical settings.

### Experimental study

In the current study, subject-specific mBGFT and ΔmBGFT were computed from MRI images of the unloaded and loaded knee joint and used to calculate mean strain. Similarly, Chan et al*.* [18] computed articular cartilage strain during load, which overlooked other tissue effects in the *in-situ* environment. However, the current study considered the strain at the whole tibiofemoral joint, which includes the effects of each component of all *WB-ST*, synovial fluid, and interaction among them. The percentage of ΔmBGFT of unloaded mBGFT was observed in a range of 6–15%, which was similar to previously reported studies, 5.23% ± 6.20 using MRI and 4.57% ± 10.31 using X-ray [16]. In the current study, the intra-subject variation of percentage ΔmBGFT in subject-1, subject-2, and subject-3 was 1%, 4.8%, and 1.01%, respectively. However, the absolute deviation was 0.06 mm, 0.35 mm, and 0.08 mm, respectively, which might be due to variations in segmentation or change in bone alignment during experimental load conditions. The change in bone alignment can alter the distribution of load across the region [36], which is reflected as the variations in the observed value of compressive stiffness [27]. However, in the current study, FEM analysis incorporates the subject-specific change in force vector due to the change in alignment.

### Simulation Study

The current study developed a method to estimate subject-specific stiffness for FEM and proposed a generalized mathematical model. The FEM was used as an efficient tool for evaluation of joint disorder [1], stress–strain distribution at articulating surface [1, 2], and estimation of body-weight for the onset of osteoarthritis (*OA*) [3] and optimization of implant selection [4]; however, previous studies [2, 3, 6, 7, 9, 37] has not used subject-specific material properties for FEM. Thus, a method to estimate subject-specific mechanical properties for FEM is proposed.

The average stiffness of the soft-tissues observed in the current study was 2.45 ± 0.13 MPa, for healthy subjects. Further, intra-subject variability in the estimated stiffness of two subjects was observed as 0.27 MPa and 0.12 MPa, respectively, within acceptable limits for FEM analysis because such small variation is trivial to yield any significant difference in results outcome.

The uniform mesh size may not be suited to the knee joint's complex geometry during FEM. Thus an adaptive convergence method was deployed for the refinement of mesh and to measure convergence error. The absolute convergence error was observed less than 1.02% for all the cases may depict further refinement of mesh have not significant change in the results.

The High computation time for the simulation studies restricts the use of this in individual data for clinical use, whereas the proposed generalized mathematical model is very handy and can even be used at a console and be useful in clinical practice.

### Mathematical model

In the previous report, Butz et al. [38] estimated the subject-specific cartilage material properties using mathematical formula and stress–strain obtained from DENSE-FSE *MR* images. However, the mathematical equation used [38] did not consider the curvature shape of the tibiofemoral interaction region. In the ideal case of strain–stiffness–stress relationship (1), where contact region is a plain surface and area of contact remains constant under load conditions (Additional file 1: Fig. S2a), coefficient 'a' is denoted as stress and coefficient '*b*' as '− 1'. However, in the curvature shape contact region as in the tibiofemoral joint, the contact area depends on the load and the stiffness of the tibiofemoral joint (Additional file 1: Fig. S2b). It has been observed that the inter-subject variation in estimated CCS was highly dependent on the scaling coefficient ‘*a*’. The scaling coefficient ‘*a*’ was derived from mean stress at the tibiofemoral joint, and it incorporates subject-specific features. Inter-subject variability in this scaling coefficient was observed because of inter-subject variability in tibiofemoral contact-area and anatomy. In contrast, intra-subject variability was observed because of variability in the orientation of bone and change in force vector during repeat scan.

Whereas the power coefficient ‘*b*’ was observed as a slightly lower negative value than ‘− 1’ because of the curvature shape of the tibiofemoral contact surface. Curvature shape changes the contact area with a change in stiffness; that is, more is the stiffness correspondingly lesser is the contact area. However, intra-subject variability in the power coefficient was observed (Table 2). This could be possible because of variability in the contact region due to orientation change during a repeat scan. The proposed generalized model (2) can estimate CCS using ΔmBGFT and mBGFT obtained by MRI images with the loading device. This study could also be extended to other imaging modalities such as standing X-ray [16] and standing MRI. However, for appropriate FEM analysis, it is recommended to drive the subject-specific mathematical equation to estimate the CCS of the tibiofemoral joint.

### Clinical perspectives

Previous studies about biomechanical properties of individual tissues of the knee joint such as cartilage [24, 25, 27–32], and meniscus [32] may be useful for the development of tissue replacement biomaterial. However, understanding the knee dynamic by FEM modeling, CCS that is collective response complex interactions of all WB-ST and synovial fluid may be more appropriate.

The load distribution among the knee compartments depends on bone alignment, structure, and material properties. Therefore, a pre-surgical study might help clinicians to understand the joint’s loading pattern, thus minimizing post-surgery adversity [38]. In addition, a pre-surgical CCS evaluation may provide the best-suited customized material properties for an individual specific knee joint.

Further, load distribution studies are also important in partial knee replacement surgery. The pre-surgery evaluation of CCS may provide the whole joint’s stiffness; further, the partial volume material properties could be changed in the FEM, which may provide load distribution patterns for surgical planning.

The subject-specific model might help longitudinal studies of the subject, evaluating the effects of exercise, aging effect, or disease progression. From a clinical perspective, combined stiffness of the tibiofemoral joint may serve as an additional indicator of the joint's health.

### Limitations

The proposed method could estimate the CCS of the tibiofemoral joint using FEM, which represented a simplified model of the complex knee joint. Further, stiffness of each knee joint’s component such as cartilage, meniscus, ligament, and synovial fluid, and their *inter-*and *intra-* component variability could only be accessed by an increase in the number of stiffness values for each compartment or component in FEM to develop a mathematical model; but, it increases the computation complexity; hence, it is not possible in routine clinical settings. Additionally, FEM is used largely for biomechanical studies. Nevertheless, simulation results may depart from realistic conditions, especially in complex structures such as knee joints. Furthermore, this study did not include subjects with osteoarthritis or patients with any other knee disease, which could show the variability between healthy and degraded soft-tissues. Thirdly, the effect of muscle tone on the bone gap is not considered in this study, which could be a further study with assessing the effect of muscle tone on knee joint health. Besides, the present model is derived from a small set of healthy clinical data; thus, the values of coefficients in the proposed generalized model for stress–strain of knee joint are subjected to further verification and experimentation with a larger dataset and across various pathological conditions.

## Conclusions

The method proposed in the current study to estimate the combined compressive stiffness of the tibiofemoral joint is a novel way to identify subject-specific biomechanical information for finite-element modeling. The study also provides a generalized model for the tibiofemoral joint's stiffness that could be used further in simulations and clinical studies with imaging modalities such as MRI, X-ray, and *CT*. In addition, such analysis might enhance the efficacy of implant design optimization and biomechanics for subject-specific studies.

## Supplementary Information

**Additional file 1.** **Figure S1**. Shows a schematic diagram of the functioning of the axial knee loading device. **Figure S2**. Effect on contact region during load with the shape of contact area, a) shows contact area remain same under load if contact region shape is plain surface, b) shows the increase in the area of contact region under applied load if contact region shape is curvature.

## Data Availability

The datasets generated and analyzed during the current study are not publicly available but may be available from the corresponding author on reasonable request.

## Abbreviations

ΔmBGFT: Difference of mBGFT of unloaded and loaded condition
3D: Three dimensional
50%BW: Fifty percentage of body-weight
AL: Augmented Lagrange
CAD: Computer-aided-design
CT: Computed Tomography
CCS: Combined-compressive-stiffness
DENSE-FSE: Displacement encoding with simulated echoes- Fast spin echo
DICOM: Digital imaging and communications in medicine
FEM: Finite-element-modeling
FSPGR: 3D-Fast spoiled gradient echo
IE: Isotropic elastic
IEC: Institutional Ethics Committee
mBGFT: Mean tibiofemoral bone gap
MPC: Multi-point constraint
MRI: Magnetic resonance imaging
PCG: Preconditioned conjugate gradients
R^2^: Goodness of fitting
RoI: Region of interest
TE: Echo time
TET10: 10 Nodes tetrahedral element
TR: Repetition time
WB-ST: Weight-bearing soft-tissue
