# Supplementary material for: Model for in-vivo estimation of stiffness of tibiofemoral joint using MR imaging and FEM analysis
Source: J Transl Med. 2021 Jul 19;19:310. doi: 10.1186/s12967-021-02977-1 (PMC8287773; doi:10.1186/s12967-021-02977-1)
Supplement: Supplementary file 1 — Additional file 1. Figure S1. Shows a schematic diagram of the functioning of the axial knee loading device. Figure S2. Effect on contact region during load with the shape of contact area, a) shows contact area remain same under load if contact region shape is plain surface, b) shows the increase in the area of contact region under applied load if contact region shape is curvature. [file 12967_2021_2977_MOESM1_ESM.docx]

Model for *in-vivo* Estimation of Stiffness of Tibiofemoral joint using MR Imaging and FEM Analysis

Sandeep Panwar Jogi, Rafeek Thaha, Sriram Rajan, Vidur Mahajan, Vasantha Kumar Venugopal, Anup Singh, Amit Mehndiratta

**SUPPLEMENTARY MATERIAL**

**Section 1: Axial Knee Loading Device**

The axial loading device (Indian Patent number: 201911021295) used in the current study consists of three main components 1) Pelvic or waist belt, 2) Elastic String, 3) Calf-rest, and 4) Foot-rest Plate. A schematic diagram of the device is presented in Supplementary figure 1.

The waist belt or pelvic belt (1) is wrapped around the waist of a subject (6) and firm contact with the hip region. The waist belt consists of a non-elastic string (1a) that is further attached with an elastic string (2). Elastic string (2) also wraps around the foot-rest plate (4). The Foot-rest plate is allowed to slide in the axial direction against the calf-rest (3) with the help of a telescopic-channel slider (5). The non-elastic string (1a) length is adjusted in such a manner to determine the stretched length of the elastic string (2); which is pre-calibrated with the load. So that the pre-determined load could be administered on a joint between the foot-rest plate (4) and waist belt (1) with the help of elastic string (2).

**
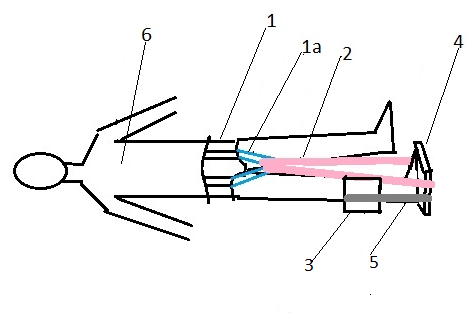
**

*SUPPLEMENTARY FIGURE 1. Shows a schematic diagram of the functioning of the axial knee loading device.*

**Section 2: 3D geometry optimization**

1. ***Rough to Smooth 3D-Surface Geometry:*** Developed 3D-surface geometry was imported in 3-MATIC Research 12.0 (Materialise NV, Leuven, Belgium) for further post-processing and optimization, such as wall smoothening, removal of unwanted spikes, etc. Wrapping operation was performed with protecting a thin wall and kept the smallest details enacted as 0.4 mm. The smoothening operation was performed gradually, with 70 iterations, using the Laplacian first-order method with a small smoothing factor [7] of 0.3. The transformation from rough to finished 3D-surface geometry after the smoothing operation is shown in Fig. 2c, and Fig. 2d.
2. **3D-Surface to solid modeling:** The final models were imported as STL file of each component as femur, femoral-cartilage, tibia, tibial-cartilage, and meniscus in Ansys 18.1 Workbench (Ansys Inc., Canonsburg, Pennsylvania, United States) for further optimization and FEA.

***3D-Surface Geometry to Solid 3D-Model and Assembly of Components:*** Static Structural Module of Ansys 18.1 Workbench was used for further processing. Surface geometries were imported in SpaceClaim via Ansys 18.1 to assemble in order of their respective coordinate space, as shown in Fig. 2e. The surface geometry assembled model was optimized by checking facets of error and overlapping. Finally, all surface geometries were converted into solid geometries using the algorithm of SpaceClaim to import in Ansys Mechanical.

**Section 3: Finite element modelling**

1. ***Assigning Contacts and Contact behavior*:** Contacts and contacts behavior of components interfaces were assigned in Ansys Mechanical Workbench. Six contacts were assigned for the knee joint interface. Out of these six contacts, three were assigned as bonded, and three were assigned as frictionless contacts. Contacts at femur with femoral-cartilage, tibia with tibial-cartilage, and tibia with meniscus were assigned as bonded contact. The Multi-Point Constraint (MPC) contact formulation was used for the solution in bonded contacts, as shown in Fig. 3a, Fig. 3b, and Fig. 3c. Contact at femoral-cartilage with tibial-cartilage, femoral-cartilage with the meniscus, and tibial-cartilage with meniscus were assigned as frictionless contact, as shown in Fig. 3d, Fig. 3e, and Fig. 3f. The Augmented Lagrange (AL) formulation was used for all frictionless contacts. Pinball radius for all contacts was kept at 50% more than that is obtained by initial contact information analysis to avoid any error due to overlap and penetration of surfaces.
2. ***Meshing Parameters and Analysis Settings:*** Mesh parameters were defined in Ansys Mechanical Workbench. Each component of the model meshed with coarse size and TET10 type elements. TET10 displays 10-nodes connecting volume to tetrahedral mesh. TET10 configuration provides an enhanced formulation for better fitting and less computational complexity [35, 36]. Meshing was done with element size 4.00 mm for all bodies with Coarse setting, fast transition, and medium level smoothening. Additional surface meshing was applied for all soft tissues with element size 1.00 mm. For analysis, Large Deflection was kept on for incorporating large deflection during the simulations.


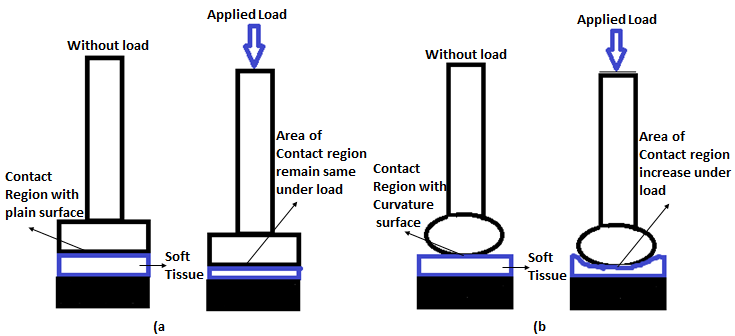


*SUPPLEMENTARY FIGURE 2. Effect on contact region during load with shape of contact area, a) shows contact area remain same under load if contact region shape is plain surface, b) shows the increase in area of contact region under applied load if contact region shape is curvature.*
